# 4-Eth­oxy-*N*-(4-eth­oxy­phen­yl)-*N*-(4-nitro­phen­yl)aniline

**DOI:** 10.1107/S1600536813023386

**Published:** 2013-08-23

**Authors:** Ming Kong, Fu-Ying Hao, Dan-Dan Li, Jie-Ying Wu

**Affiliations:** a Department of Chemistry, Anhui University, Hefei 230039, People’s Republic of China, Key Laboratory of Functional Inorganic Materials Chemistry, Hefei 230039, People’s Republic of China

## Abstract

In the title mol­ecule, C_22_H_22_N_2_O_4_, the eth­oxy­phenyl rings are oriented at dihedral angles of 69.31 (13) and 75.90 (13)° to the nitro­phenyl ring and are twisted to each other, making a dihedral angle of 78.55 (13)°. In the crystal, weak C—H⋯O hydrogen bonds and C—H⋯π inter­action link the mol­ecules into a three-dimensional supra­molecular architecture.

## Related literature

For applications of tri­phenyl­amine derivatives, see: Liu *et al.* (2012 ▶). For related compounds, see: Wang *et al.* (2011 ▶); Gudeika *et al.* (2012 ▶).
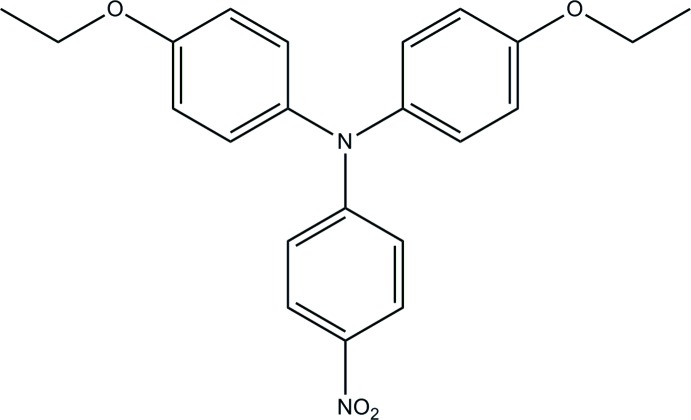


## Experimental

### 

#### Crystal data

C_22_H_22_N_2_O_4_
*M* *_r_* = 378.42
Monoclinic, 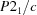

*a* = 10.926 (5) Å
*b* = 18.380 (5) Å
*c* = 10.345 (5) Å
β = 107.998 (5)°
*V* = 1975.8 (14) Å^3^
*Z* = 4
Mo *K*α radiation
μ = 0.09 mm^−1^
*T* = 298 K
0.30 × 0.20 × 0.20 mm

#### Data collection

Bruker SMART 1000 CCD area-detector diffractometer
13718 measured reflections
3456 independent reflections
1884 reflections with *I* > 2σ(*I*)
*R*_int_ = 0.050

#### Refinement

*R*[*F* ^2^ > 2σ(*F* ^2^)] = 0.054
*wR*(*F* ^2^) = 0.178
*S* = 0.98
3456 reflections
255 parameters
H-atom parameters constrained
Δρ_max_ = 0.18 e Å^−3^
Δρ_min_ = −0.17 e Å^−3^

### 

Data collection: *SMART* (Bruker, 2007 ▶); cell refinement: *SAINT* (Bruker, 2007 ▶); data reduction: *SAINT*; program(s) used to solve structure: *SHELXTL* (Sheldrick, 2008 ▶); program(s) used to refine structure: *SHELXTL*; molecular graphics: *SHELXTL*; software used to prepare material for publication: *SHELXTL*.

## Supplementary Material

Crystal structure: contains datablock(s) I, Global. DOI: 10.1107/S1600536813023386/xu5734sup1.cif

Structure factors: contains datablock(s) I. DOI: 10.1107/S1600536813023386/xu5734Isup2.hkl

Supplementary material file. DOI: 10.1107/S1600536813023386/xu5734Isup3.cml

Additional supplementary materials:  crystallographic information; 3D view; checkCIF report

## Figures and Tables

**Table 1:** Hydrogen-bond geometry (Å, °) *Cg*2 is the centroid of the C7–C12 benzene ring.

*D*—H⋯*A*	*D*—H	H⋯*A*	*D*⋯*A*	*D*—H⋯*A*
C7—H7⋯O3^i^	0.93	2.56	3.371 (5)	146
C14—H14*B*⋯O4^ii^	0.96	2.55	3.458 (4)	158
C17—H17⋯*Cg*2^iii^	0.93	2.78	3.700 (4)	173

Symmetry codes: (i) 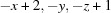; (ii) 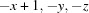; (iii) 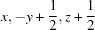.

**Fig. 1.**
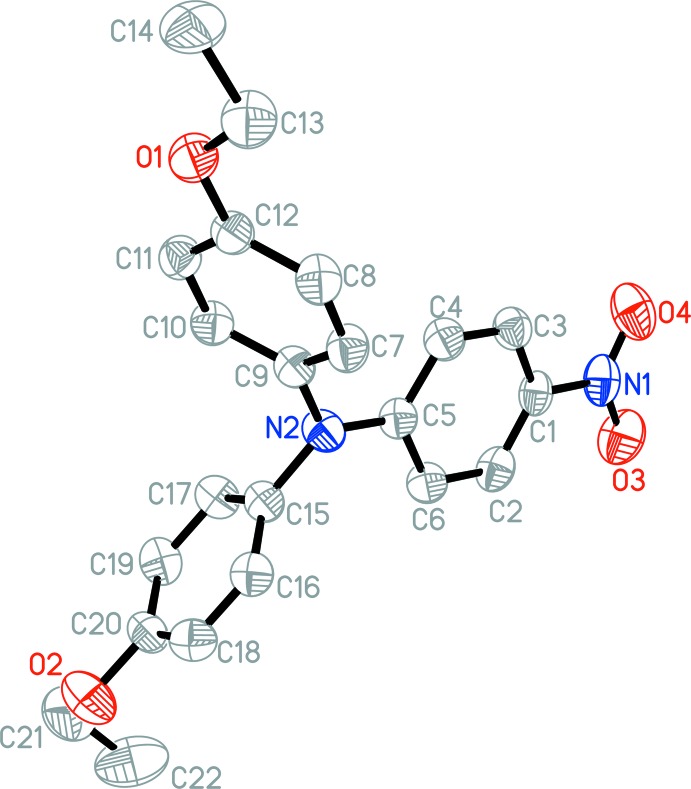
The molecular structure of the title compound (I) showing 30% probability displacement ellipsoids.

## supplementary crystallographic information

### 1. Comment

As optical functional materials, triphenylamine derivative have attracted considerable attention, for their fluorescent characters, photostabilities and easy modification (Liu *et al.*, 2012). Besides, the strong ability of electron delocalization in the title compound forms conjugated system which is one of the most excellent properties in two photon absorption materials field. Recent years many research groups choose triphenylamine as molecule core to synthesize a series of compounds (Wang *et al.*, 2011; Gudeika *et al.*, 2012). The length of the two nitrogen oxygen bonds in the crystal structure is nearly identical comparing their bond length data of 1.232 (3) and 1.229 (3) Å. This shows that the two bonds are intervenient between nitrogen oxygen single bond and nitrogen oxygen double bond. Also, the introduction of nitro group transforms the benzene ring plane generatting a dihedral angle of 2.8 (7)° being defined by the two planes of plane1 (C1, C2, C3) and plane2 (C4, C5, C6).

### 2. Experimental

The intermediate 1-ethoxy-4-iodobenzene was synthesized by mixing 4-iodophenol (110 g, 0.5 mol) with bromoethane (218 g, 2 mol) in methanol (250 ml) in the presence of NaOH (40 g, 1 mol). The mixture was heated to reflux for 12 h. The solution was cooled to room temperature. White solid appeared when poured into a large amount of ice water. The solid was purified by 20% NaOH solution. The target product was obtained by the mixture of 4-nitroaniline (1.97 g, 15 mmol), 1-ethoxy-4-iodobenzene (10 g, 40 mmol), K_2_CO_3_ (13.8 g, 100 mmol), a few of *L*-proline and CuI in DMSO at 100 degrees celsius for 24 h. The mixture was washed with plenty of water and extracted by dichloromethane. The combined organic layer was dried over anhydrous MgSO_4_ and concentrated using a rotary evaporator. The residue was purified by column chromatography. 1H NMR: (400 Hz, DMSO-d_6_), d(p.p.m.):8.10 (d, 2H), 7.32 (d, 2H), 7.02 (d, 4H), 6.71 (d, 4H), 4.03 (q, 4H), 1.35 (t, 6H)

### 3. Refinement

All hydrogen atoms were placed in geometrically idealized positions and constrained to ride on their parent atoms with C—H = 0.93–0.97 Å, *U*_iso_(H) = 1.2*U*_eq_(C) or 1.5*U*_eq_(C).

### Crystal data

**Table:**

C_22_H_22_N_2_O_4_	*F*(000) = 800
*M**_r_* = 378.42	*D*_x_ = 1.272 Mg m^−^^3^
Monoclinic, *P*2_1_/*c*	Mo *K*α radiation, λ = 0.71069 Å
Hall symbol: -P 2ybc	Cell parameters from 1657 reflections
*a* = 10.926 (5) Å	θ = 2.3–19.5°
*b* = 18.380 (5) Å	µ = 0.09 mm^−^^1^
*c* = 10.345 (5) Å	*T* = 298 K
β = 107.998 (5)°	Block, red
*V* = 1975.8 (14) Å^3^	0.30 × 0.20 × 0.20 mm
*Z* = 4	

### Data collection

**Table:**

Bruker SMART 1000 CCD area-detector diffractometer	1884 reflections with *I* > 2σ(*I*)
Radiation source: fine-focus sealed tube	*R*_int_ = 0.050
Graphite monochromator	θ_max_ = 25.0°, θ_min_ = 2.0°
phi and ω scans	*h* = −12→12
13718 measured reflections	*k* = −20→21
3456 independent reflections	*l* = −12→12

### Refinement

**Table:**

Refinement on *F*^2^	Primary atom site location: structure-invariant direct methods
Least-squares matrix: full	Secondary atom site location: difference Fourier map
*R*[*F*^2^ > 2σ(*F*^2^)] = 0.054	Hydrogen site location: inferred from neighbouring sites
*wR*(*F*^2^) = 0.178	H-atom parameters constrained
*S* = 0.98	*w* = 1/[σ^2^(*F*_o_^2^) + (0.1*P*)^2^] where *P* = (*F*_o_^2^ + 2*F*_c_^2^)/3
3456 reflections	(Δ/σ)_max_ < 0.001
255 parameters	Δρ_max_ = 0.18 e Å^−^^3^
0 restraints	Δρ_min_ = −0.17 e Å^−^^3^

### Special details

**Table:**

Geometry. All e.s.d.'s (except the e.s.d. in the dihedral angle between two l.s. planes) are estimated using the full covariance matrix. The cell e.s.d.'s are taken into account individually in the estimation of e.s.d.'s in distances, angles and torsion angles; correlations between e.s.d.'s in cell parameters are only used when they are defined by crystal symmetry. An approximate (isotropic) treatment of cell e.s.d.'s is used for estimating e.s.d.'s involving l.s. planes.
Refinement. Refinement of *F*^2^ against ALL reflections. The weighted *R*-factor *wR* and goodness of fit *S* are based on *F*^2^, conventional *R*-factors *R* are based on *F*, with *F* set to zero for negative *F*^2^. The threshold expression of *F*^2^ > σ(*F*^2^) is used only for calculating *R*-factors(gt) *etc*. and is not relevant to the choice of reflections for refinement. *R*-factors based on *F*^2^ are statistically about twice as large as those based on *F*, and *R*- factors based on ALL data will be even larger.

### Fractional atomic coordinates and isotropic or equivalent isotropic displacement parameters (Å^2^)

**Table:**

	*x*	*y*	*z*	*U*_iso_*/*U*_eq_	
N2	0.91897 (19)	0.16945 (11)	0.28936 (19)	0.0673 (6)	
O1	0.56239 (17)	0.18864 (10)	−0.23010 (17)	0.0785 (6)	
C9	0.8292 (2)	0.17241 (14)	0.1544 (2)	0.0607 (7)	
C8	0.7178 (3)	0.11519 (15)	−0.0584 (3)	0.0708 (8)	
H8	0.7016	0.0741	−0.1136	0.085*	
C1	0.8614 (3)	0.04099 (13)	0.5958 (2)	0.0635 (7)	
C15	1.0222 (2)	0.22159 (14)	0.3220 (2)	0.0609 (7)	
C7	0.8063 (3)	0.11222 (15)	0.0714 (3)	0.0704 (7)	
H7	0.8504	0.0692	0.1022	0.084*	
C12	0.6541 (2)	0.17944 (15)	−0.1048 (2)	0.0633 (7)	
C6	0.9971 (3)	0.11691 (14)	0.5137 (2)	0.0661 (7)	
H6	1.0756	0.1404	0.5282	0.079*	
N1	0.8397 (3)	−0.00099 (13)	0.7052 (3)	0.0829 (7)	
C5	0.9009 (2)	0.12531 (13)	0.3896 (2)	0.0585 (6)	
C17	1.0181 (3)	0.28165 (15)	0.4004 (2)	0.0708 (8)	
H17	0.9496	0.2878	0.4349	0.085*	
C10	0.7672 (2)	0.23642 (15)	0.1048 (3)	0.0674 (7)	
H10	0.7842	0.2779	0.1590	0.081*	
C3	0.7654 (3)	0.04742 (14)	0.4739 (3)	0.0697 (7)	
H3	0.6872	0.0239	0.4612	0.084*	
C4	0.7844 (3)	0.08837 (14)	0.3707 (2)	0.0687 (7)	
H4	0.7196	0.0916	0.2878	0.082*	
C19	1.1156 (3)	0.33271 (15)	0.4278 (3)	0.0744 (8)	
H19	1.1132	0.3731	0.4810	0.089*	
C20	1.2164 (3)	0.32350 (16)	0.3758 (3)	0.0708 (8)	
O4	0.7323 (3)	−0.02692 (13)	0.6884 (2)	0.1098 (8)	
C2	0.9775 (3)	0.07440 (14)	0.6150 (2)	0.0709 (8)	
H2	1.0433	0.0684	0.6965	0.085*	
O2	1.3142 (2)	0.37328 (13)	0.3914 (2)	0.1142 (8)	
C11	0.6805 (2)	0.23998 (15)	−0.0236 (3)	0.0689 (7)	
H11	0.6395	0.2837	−0.0555	0.083*	
O3	0.9283 (3)	−0.00806 (12)	0.8122 (2)	0.1142 (8)	
C18	1.2193 (3)	0.26372 (16)	0.2976 (3)	0.0738 (8)	
H18	1.2874	0.2575	0.2625	0.089*	
C16	1.1235 (3)	0.21351 (15)	0.2711 (2)	0.0675 (7)	
H16	1.1265	0.1732	0.2179	0.081*	
C13	0.5245 (3)	0.12818 (17)	−0.3152 (3)	0.0908 (9)	
H13A	0.4952	0.0896	−0.2679	0.109*	
H13B	0.5966	0.1101	−0.3418	0.109*	
C14	0.4178 (3)	0.15093 (18)	−0.4383 (3)	0.1043 (11)	
H14A	0.3416	0.1603	−0.4134	0.157*	
H14B	0.4009	0.1128	−0.5049	0.157*	
H14C	0.4424	0.1943	−0.4757	0.157*	
C21	1.3504 (4)	0.4187 (2)	0.5060 (5)	0.1273 (14)	
H21A	1.2735	0.4357	0.5246	0.153*	
H21B	1.3948	0.4609	0.4861	0.153*	
C22	1.4317 (4)	0.3834 (3)	0.6241 (4)	0.1555 (19)	
H22A	1.3818	0.3499	0.6583	0.233*	
H22B	1.4696	0.4191	0.6925	0.233*	
H22C	1.4984	0.3575	0.6012	0.233*	

### Atomic displacement parameters (Å^2^)

**Table:**

	*U*^11^	*U*^22^	*U*^33^	*U*^12^	*U*^13^	*U*^23^
N2	0.0709 (14)	0.0801 (15)	0.0448 (12)	−0.0157 (12)	0.0088 (11)	0.0000 (10)
O1	0.0823 (13)	0.0837 (13)	0.0582 (11)	−0.0070 (10)	0.0055 (10)	0.0034 (10)
C9	0.0660 (16)	0.0682 (17)	0.0454 (14)	−0.0081 (13)	0.0138 (12)	0.0001 (13)
C8	0.0888 (19)	0.0678 (18)	0.0499 (16)	−0.0088 (15)	0.0131 (14)	−0.0086 (13)
C1	0.089 (2)	0.0510 (15)	0.0502 (16)	0.0035 (14)	0.0212 (15)	0.0016 (12)
C15	0.0668 (16)	0.0706 (17)	0.0416 (13)	−0.0087 (14)	0.0111 (12)	0.0006 (12)
C7	0.0844 (19)	0.0645 (17)	0.0557 (17)	−0.0031 (14)	0.0122 (14)	0.0015 (13)
C12	0.0649 (16)	0.0737 (19)	0.0482 (15)	−0.0078 (14)	0.0129 (13)	0.0067 (14)
C6	0.0709 (17)	0.0708 (17)	0.0506 (16)	−0.0037 (13)	0.0100 (14)	−0.0025 (13)
N1	0.122 (2)	0.0627 (15)	0.0669 (18)	0.0024 (15)	0.0329 (18)	0.0051 (13)
C5	0.0676 (17)	0.0603 (15)	0.0456 (14)	−0.0015 (13)	0.0145 (13)	−0.0012 (12)
C17	0.0784 (18)	0.081 (2)	0.0578 (16)	−0.0091 (15)	0.0283 (14)	−0.0113 (14)
C10	0.0750 (18)	0.0654 (17)	0.0573 (17)	−0.0058 (14)	0.0140 (14)	−0.0075 (13)
C3	0.0780 (19)	0.0664 (17)	0.0628 (18)	−0.0064 (14)	0.0192 (15)	0.0030 (13)
C4	0.0759 (19)	0.0733 (18)	0.0509 (15)	−0.0048 (14)	0.0110 (13)	0.0032 (13)
C19	0.096 (2)	0.0728 (18)	0.0534 (16)	−0.0129 (16)	0.0213 (15)	−0.0118 (13)
C20	0.0671 (18)	0.081 (2)	0.0609 (17)	−0.0175 (15)	0.0143 (14)	0.0071 (15)
O4	0.149 (2)	0.0996 (17)	0.0863 (16)	−0.0303 (16)	0.0442 (16)	0.0067 (12)
C2	0.089 (2)	0.0679 (17)	0.0475 (15)	0.0074 (15)	0.0095 (14)	0.0005 (13)
O2	0.1123 (17)	0.1195 (19)	0.1136 (19)	−0.0450 (16)	0.0391 (15)	−0.0177 (15)
C11	0.0712 (17)	0.0653 (17)	0.0660 (17)	−0.0010 (14)	0.0148 (14)	0.0018 (14)
O3	0.142 (2)	0.1228 (19)	0.0680 (15)	0.0173 (16)	0.0181 (15)	0.0345 (13)
C18	0.0690 (18)	0.090 (2)	0.0643 (17)	0.0004 (16)	0.0228 (14)	−0.0049 (15)
C16	0.0748 (17)	0.0720 (18)	0.0555 (15)	−0.0003 (15)	0.0197 (14)	−0.0019 (13)
C13	0.108 (2)	0.092 (2)	0.0624 (19)	−0.0062 (19)	0.0113 (17)	−0.0076 (17)
C14	0.092 (2)	0.131 (3)	0.067 (2)	−0.009 (2)	−0.0090 (17)	−0.0045 (19)
C21	0.128 (3)	0.088 (3)	0.159 (4)	−0.029 (2)	0.035 (3)	−0.046 (3)
C22	0.112 (3)	0.218 (5)	0.118 (4)	0.022 (3)	0.008 (3)	−0.046 (4)

### Geometric parameters (Å, º)

**Table:**

N2—C5	1.378 (3)	C10—H10	0.9300
N2—C9	1.439 (3)	C3—C4	1.373 (3)
N2—C15	1.439 (3)	C3—H3	0.9300
O1—C12	1.382 (3)	C4—H4	0.9300
O1—C13	1.399 (3)	C19—C20	1.377 (4)
C9—C7	1.375 (3)	C19—H19	0.9300
C9—C10	1.375 (3)	C20—C18	1.370 (4)
C8—C12	1.379 (4)	C20—O2	1.378 (3)
C8—C7	1.393 (4)	C2—H2	0.9300
C8—H8	0.9300	O2—C21	1.403 (4)
C1—C2	1.368 (4)	C11—H11	0.9300
C1—C3	1.373 (3)	C18—C16	1.359 (4)
C1—N1	1.449 (3)	C18—H18	0.9300
C15—C16	1.372 (3)	C16—H16	0.9300
C15—C17	1.379 (3)	C13—C14	1.496 (4)
C7—H7	0.9300	C13—H13A	0.9700
C12—C11	1.370 (3)	C13—H13B	0.9700
C6—C2	1.376 (4)	C14—H14A	0.9600
C6—C5	1.394 (3)	C14—H14B	0.9600
C6—H6	0.9300	C14—H14C	0.9600
N1—O4	1.229 (3)	C21—C22	1.426 (5)
N1—O3	1.232 (3)	C21—H21A	0.9700
C5—C4	1.402 (3)	C21—H21B	0.9700
C17—C19	1.382 (3)	C22—H22A	0.9600
C17—H17	0.9300	C22—H22B	0.9600
C10—C11	1.374 (3)	C22—H22C	0.9600
			
C5—N2—C9	122.7 (2)	C20—C19—C17	119.7 (3)
C5—N2—C15	119.98 (19)	C20—C19—H19	120.2
C9—N2—C15	116.93 (19)	C17—C19—H19	120.2
C12—O1—C13	118.8 (2)	C18—C20—C19	119.8 (3)
C7—C9—C10	118.8 (2)	C18—C20—O2	116.3 (3)
C7—C9—N2	120.9 (2)	C19—C20—O2	123.9 (3)
C10—C9—N2	120.3 (2)	C1—C2—C6	120.0 (2)
C12—C8—C7	119.6 (2)	C1—C2—H2	120.0
C12—C8—H8	120.2	C6—C2—H2	120.0
C7—C8—H8	120.2	C20—O2—C21	120.0 (3)
C2—C1—C3	120.3 (2)	C12—C11—C10	120.4 (3)
C2—C1—N1	119.6 (3)	C12—C11—H11	119.8
C3—C1—N1	120.0 (3)	C10—C11—H11	119.8
C16—C15—C17	119.3 (2)	C16—C18—C20	120.4 (3)
C16—C15—N2	120.3 (2)	C16—C18—H18	119.8
C17—C15—N2	120.4 (2)	C20—C18—H18	119.8
C9—C7—C8	120.6 (3)	C18—C16—C15	120.8 (3)
C9—C7—H7	119.7	C18—C16—H16	119.6
C8—C7—H7	119.7	C15—C16—H16	119.6
C11—C12—C8	119.6 (2)	O1—C13—C14	108.4 (3)
C11—C12—O1	115.5 (2)	O1—C13—H13A	110.0
C8—C12—O1	124.9 (2)	C14—C13—H13A	110.0
C2—C6—C5	120.9 (3)	O1—C13—H13B	110.0
C2—C6—H6	119.5	C14—C13—H13B	110.0
C5—C6—H6	119.5	H13A—C13—H13B	108.4
O4—N1—O3	122.5 (3)	C13—C14—H14A	109.5
O4—N1—C1	118.4 (3)	C13—C14—H14B	109.5
O3—N1—C1	119.0 (3)	H14A—C14—H14B	109.5
N2—C5—C6	121.1 (2)	C13—C14—H14C	109.5
N2—C5—C4	121.0 (2)	H14A—C14—H14C	109.5
C6—C5—C4	117.9 (2)	H14B—C14—H14C	109.5
C15—C17—C19	120.1 (3)	O2—C21—C22	113.1 (3)
C15—C17—H17	120.0	O2—C21—H21A	109.0
C19—C17—H17	120.0	C22—C21—H21A	109.0
C11—C10—C9	120.9 (2)	O2—C21—H21B	109.0
C11—C10—H10	119.5	C22—C21—H21B	109.0
C9—C10—H10	119.5	H21A—C21—H21B	107.8
C1—C3—C4	120.3 (3)	C21—C22—H22A	109.5
C1—C3—H3	119.8	C21—C22—H22B	109.5
C4—C3—H3	119.8	H22A—C22—H22B	109.5
C3—C4—C5	120.4 (2)	C21—C22—H22C	109.5
C3—C4—H4	119.8	H22A—C22—H22C	109.5
C5—C4—H4	119.8	H22B—C22—H22C	109.5
			
C5—N2—C9—C7	−63.6 (3)	N2—C15—C17—C19	−177.9 (2)
C15—N2—C9—C7	124.0 (3)	C7—C9—C10—C11	2.2 (4)
C5—N2—C9—C10	118.2 (3)	N2—C9—C10—C11	−179.6 (2)
C15—N2—C9—C10	−54.2 (3)	C2—C1—C3—C4	−1.0 (4)
C5—N2—C15—C16	113.0 (3)	N1—C1—C3—C4	178.7 (2)
C9—N2—C15—C16	−74.3 (3)	C1—C3—C4—C5	−1.4 (4)
C5—N2—C15—C17	−69.5 (3)	N2—C5—C4—C3	−176.8 (2)
C9—N2—C15—C17	103.2 (3)	C6—C5—C4—C3	2.2 (4)
C10—C9—C7—C8	−2.8 (4)	C15—C17—C19—C20	0.3 (4)
N2—C9—C7—C8	179.0 (2)	C17—C19—C20—C18	−0.1 (4)
C12—C8—C7—C9	1.1 (4)	C17—C19—C20—O2	176.4 (2)
C7—C8—C12—C11	1.2 (4)	C3—C1—C2—C6	2.4 (4)
C7—C8—C12—O1	−178.5 (2)	N1—C1—C2—C6	−177.2 (2)
C13—O1—C12—C11	−176.0 (2)	C5—C6—C2—C1	−1.5 (4)
C13—O1—C12—C8	3.7 (4)	C18—C20—O2—C21	−154.8 (3)
C2—C1—N1—O4	175.8 (3)	C19—C20—O2—C21	28.6 (4)
C3—C1—N1—O4	−3.9 (4)	C8—C12—C11—C10	−1.8 (4)
C2—C1—N1—O3	−2.7 (4)	O1—C12—C11—C10	177.9 (2)
C3—C1—N1—O3	177.7 (2)	C9—C10—C11—C12	0.1 (4)
C9—N2—C5—C6	171.9 (2)	C19—C20—C18—C16	0.0 (4)
C15—N2—C5—C6	−15.9 (3)	O2—C20—C18—C16	−176.8 (2)
C9—N2—C5—C4	−9.1 (4)	C20—C18—C16—C15	0.0 (4)
C15—N2—C5—C4	163.1 (2)	C17—C15—C16—C18	0.3 (4)
C2—C6—C5—N2	178.2 (2)	N2—C15—C16—C18	177.8 (2)
C2—C6—C5—C4	−0.8 (4)	C12—O1—C13—C14	175.5 (2)
C16—C15—C17—C19	−0.4 (4)	C20—O2—C21—C22	79.8 (4)

### Hydrogen-bond geometry (Å, º)

Cg2 is the centroid of the C7–C12 benzene ring.

**Table:**

*D*—H···*A*	*D*—H	H···*A*	*D*···*A*	*D*—H···*A*
C7—H7···O3^i^	0.93	2.56	3.371 (5)	146
C14—H14*B*···O4^ii^	0.96	2.55	3.458 (4)	158
C17—H17···*Cg*2^iii^	0.93	2.78	3.700 (4)	173

Symmetry codes: (i) −*x*+2, −*y*, −*z*+1; (ii) −*x*+1, −*y*, −*z*; (iii) *x*, −*y*+1/2, *z*+1/2.
